# Optimization of tube voltage in X-ray dark-field chest radiography

**DOI:** 10.1038/s41598-019-45256-2

**Published:** 2019-06-18

**Authors:** Andreas P. Sauter, Jana Andrejewski, Fabio De Marco, Konstantin Willer, Lukas B. Gromann, Wolfgang Noichl, Fabian Kriner, Florian Fischer, Christian Braun, Thomas Koehler, Felix Meurer, Alexander A. Fingerle, Daniela Pfeiffer, Ernst Rummeny, Julia Herzen, Franz Pfeiffer

**Affiliations:** 1 Department of Diagnostic and Interventional Radiology, Technical University of Munich, 81675 Munich, Germany; 2 Chair of Biomedical Physics, Department of Physics and Munich School of BioEngineering, Technical University of Munich, 85748 Garching, Germany; 3 Institut für Rechtsmedizin, Ludwig-Maximilians-Universität München, 80336 Munich, Germany; 4 Philips GmbH Innovative Technologies, Research Laboratories, 22335 Hamburg, Germany

**Keywords:** Diagnosis, Medical imaging, Imaging techniques

## Abstract

Grating-based X-ray dark-field imaging is a novel imaging modality which has been refined during the last decade. It exploits the wave-like behaviour of X-radiation and can nowadays be implemented with existing X-ray tubes used in clinical applications. The method is based on the detection of small-angle X-ray scattering, which occurs e.g. at air-tissue-interfaces in the lung or bone-fat interfaces in spongy bone. In contrast to attenuation-based chest X-ray imaging, the optimal tube voltage for dark-field imaging of the thorax has not yet been examined. In this work, dark-field scans with tube voltages ranging from 60 to 120 kVp were performed on a deceased human body. We analyzed the resulting images with respect to subjective and objective image quality, and found that the optimum tube voltage for dark-field thorax imaging at the used setup is at rather low energies of around 60 to 70 kVp. Furthermore, we found that at these tube voltages, the transmission radiographs still exhibit sufficient image quality to correlate dark-field information. Therefore, this study may serve as an important guideline for the development of clinical dark-field chest X-ray imaging devices for future routine use.

## Introduction

Grating-based X-ray imaging is able to retrieve a differential-phase and a dark-field image, which visualize the magnitude of X-ray refraction and small-angle scatter, respectively^1^. Additionally, a conventional attenuation image is obtained in every image acquisition. Differential-phase and dark-field information is retrieved by generation of a fringe pattern with a very high spatial frequency via an optical grating. Attenuation, refraction and small-angle scatter modulate this pattern in such a way that their effects can be independently quantified. The relative contrast of this fringe pattern is called “(interferometric) visibility”. By placing a scattering object in the beam path, the visibility will be reduced and the dark-field image can be calculated from the ratio of reduced and original visibility (i.e., with no sample in the beam path). Introducing a second optical grating near the detector allows implementation with commonly-used X-ray detectors with large pixel sizes. Introduction of a suitable third grating near the X-ray source allows exploiting the Lau effect, which enables use of the setup with non-microfocus X-ray sources, and therefore makes routine clinical use of the technique possible^2,3^. Small-angle X-ray scattering occurs e.g. at air-tissue interfaces in the lung, or bone-fat interfaces in spongy bone. Thus, a high dark-field signal is generated by these tissues. In contrast, conventional X-ray imaging detects only the degree of X-ray attenuation, resulting in transmission radiographs.

Multiple applications for dark-field imaging have been described as results of *in-vivo* and *ex-vivo* animal studies^4–9^. Among these, imaging of the lung is of special interest, as the lung produces a strong signal due to the many interfaces between air and soft tissue^7^. Structural lung diseases (such as pulmonary emphysema or pulmonary fibrosis) reduce the signal and can be detected in early stages with clearly higher sensitivities compared to conventional radiographs in mice^10–12^. Multiple other applications of dark-field imaging were developed in experimental setups. Those include diagnosis of lung cancer, the examination of the breast and of atherosclerotic vessel changes as well as the detection of osteoporosis^5,13–15^. These initial studies are based on dark-field images obtained with small-animal scanners. Due to significant technical developments, imaging of pigs became possible during the last years^16,17^, and the first dark-field image of a human body was presented recently^18^.

X-ray imaging has been part of clinical practice for more than a century and thus, well-established protocols for all clinical setups exist. In this context, optimal tube voltages (kVp) for projection radiographs were widely examined during the last decades^19,20^. Conventional projection radiographs of the lung are performed with 120 to 130 kVp, whereas radiographs of bones are typically obtained with 70 to 80 kVp^21,22^. At these tube voltages, contrast due to absorption characteristics of the examined tissues is ideal, resulting in an optimal diagnostic value of the examination^23^. To date, most dark-field imaging is performed with lower tube voltages. For both attenuation-based and dark-field X-ray imaging, the range of suitable tube voltages is limited by the requirement that a reasonably high detector dose is achieved, while the dose delivered to the patient is limited to an acceptably low level.

For dark-field imaging, a second important limitation exists, namely that both dark-field signal generated by the imaged organ and interferometric visibility are sufficiently high. Since the decrement *δ* of the real part of the refractive index is proportional to the inverse square of photon energy^24^, the signal magnitude of the logarithmic dark-field signal decreases at least as rapidly. Furthermore, the dark-field signal magnitude is related to the value of the (projected) autocorrelation function of the sample’s electron density at the setup’s autocorrelation length *ξ*^25,26^. An increase of photon energy *E* leads to a decrease of *ξ*, where the autocorrelation function will typically achieve a higher value (if *ξ* is smaller than the characteristic length scale of the imaged sample). This finally results in further decrease of dark-field signal strength for higher photon energies.

Interferometric visibility at high X-ray energies is limited by grating manufacturing and setup length constraints: Gold is most commonly used in LIGA-manufactured^27^ X-ray attenuation gratings due to its good X-ray absorption capabilities. For photon energies just below its K-edge energy (80.7 keV), it is however nearly transparent, which leads to very poor visibility in this energy range.

Lastly, suitable inter-grating distances (yielding high visibility) for Talbot-Lau interferometers are proportional to *p*^2^*E*, where *p* is the period of the modulating grating. An efficient high-energy Talbot-Lau interferometer therefore requires gratings with small periods or a high setup length. The former is in conflict with the demand for high, strongly absorbing grating ridges, the latter is limited by spatial and technical constraints.

Until now, ideal tube voltages for dark-field thorax radiography were not examined systematically. The aim of the current study was to determine the optimal tube voltage for dark-field radiographs via evaluation of subjective and objective image quality in a series of thorax scans of a human body with different tube voltages.

## Results

**Figure 1:**
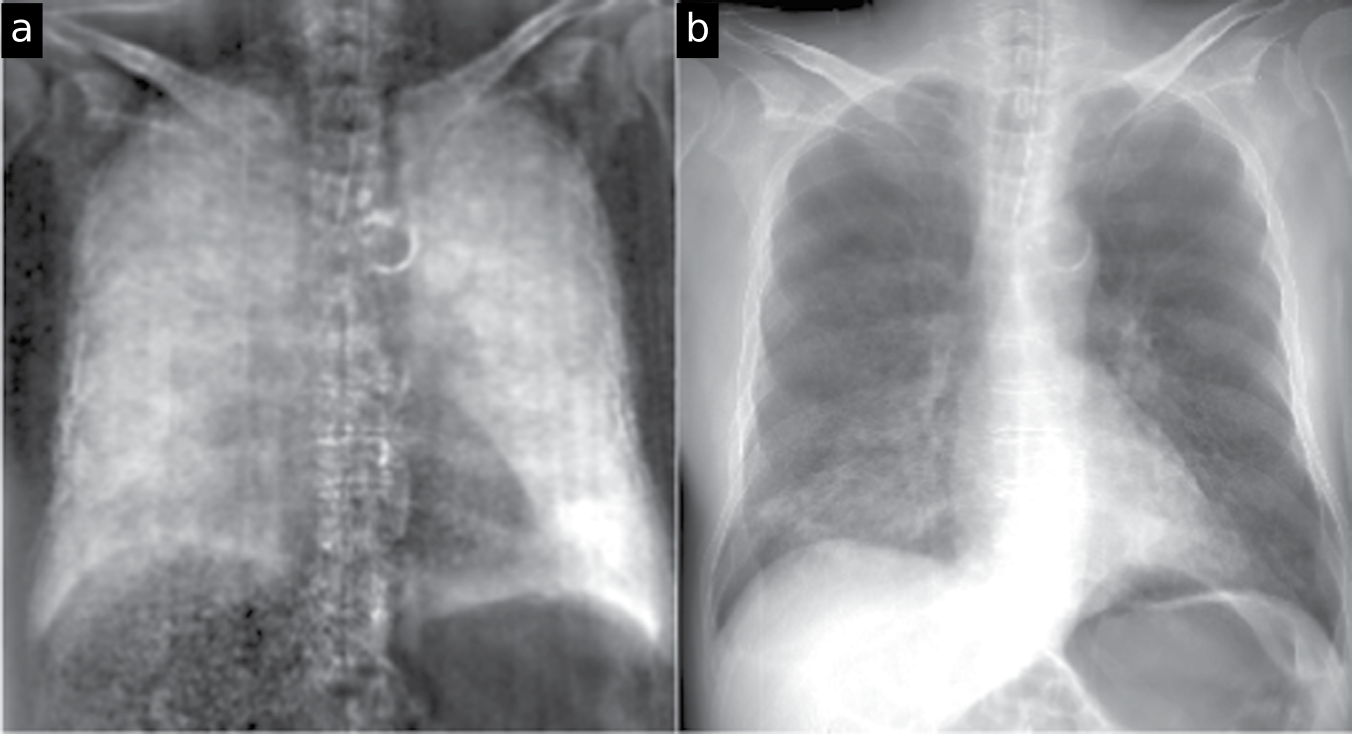
X-ray dark-field (**a**) and transmission (**b**) radiograph of a human thorax at 60kVp. The dark-field image was low-pass filtered by convolution with a 2D Gaussian kernel (*σ* = 3.2 px). In the transmission image, an infiltrate is visible in the lower left lung, which translates to a lower dark-field signal in this zone. Of all examined tube voltages (see also Fig. 2), the highest dark-field signal strength can be found at 60 kVp.

**Figure 2:**
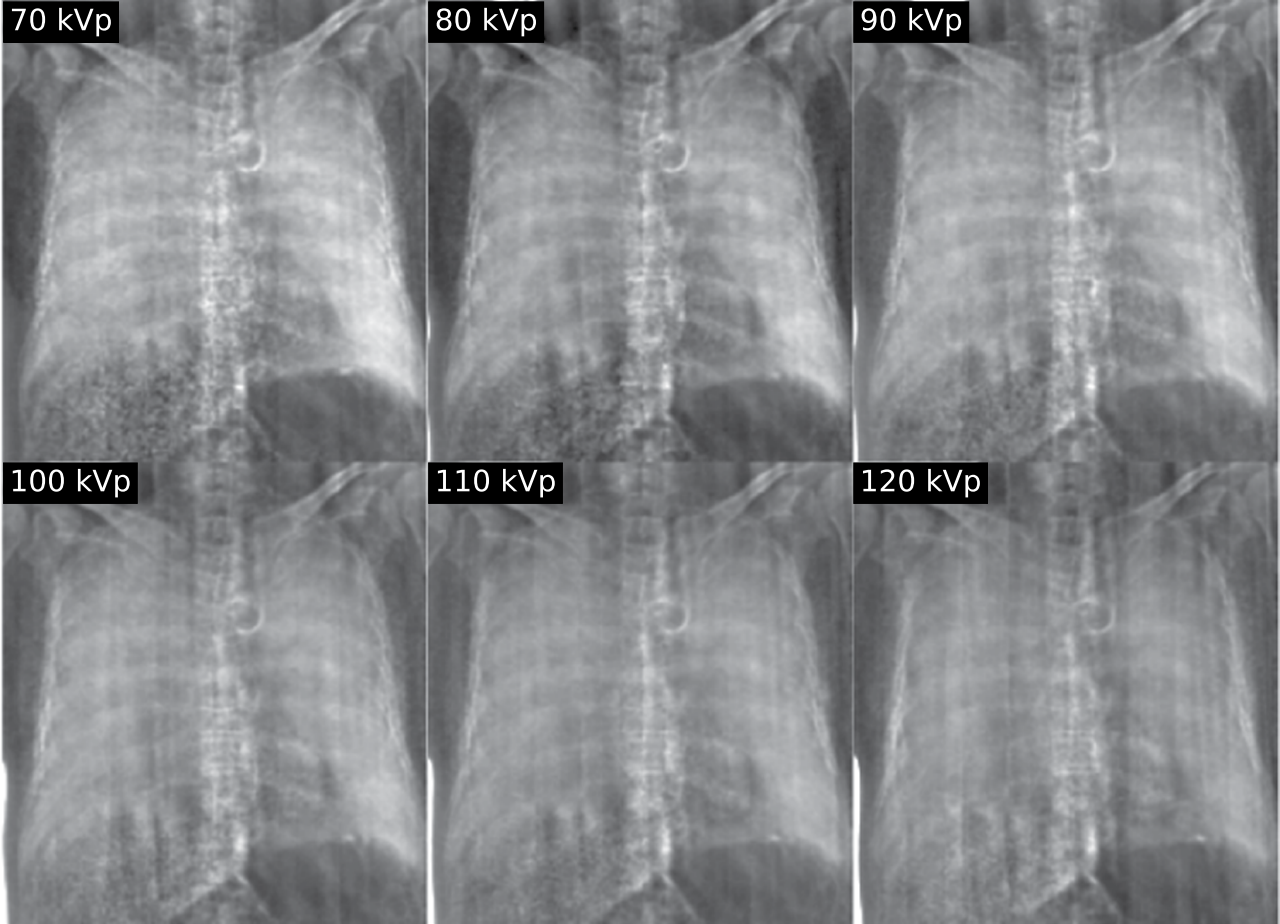
X-ray dark-field radiographs of a human thorax at the examined tube voltages. All images presented here and in Fig. 1a are shown with the same windowing and the same low-pass filter as in Fig. 1a was applied. The dark-field signal declines significantly toward higher tube voltages. Also, the image quality decreases with higher tube voltages due an increase of imaging artifacts.

We acquired X-ray dark-field and transmission thorax radiographs of one human body at tube acceleration voltages ranging from 60 to 120 kVp in 10 kVp intervals. The dark-field and transmission image of the thorax at 60kVp are shown in Fig. 1, the remaining images are shown in Fig. 2 and Supplementary Fig. S3. Image perspective is “facing the patient”, i.e. the left lung is seen on the right hand side and vice versa. Based on these images, a reader study with three readers was conducted (cf. Methods).

### Reader study

Signal strength score (opacification) for transmission radiographs showed the same median at each tube voltage in each of the six defined lung zones (cf. Methods), apart from the upper and the middle zone of the right lung. Here, the median at 120 kVp differed by 1 point (on an ordinal integer scale ranging from 0 to 5, cf. Methods) compared to the remaining tube voltages. Median signal strength score was higher for the left lung and for the upper parts of the lungs, indicating that less opacification was seen in these zones. These results reflect the autopsy findings of the examined body, as an infiltrate was present in the right lung with predominance in the right lower zone.

**Figure 3:**
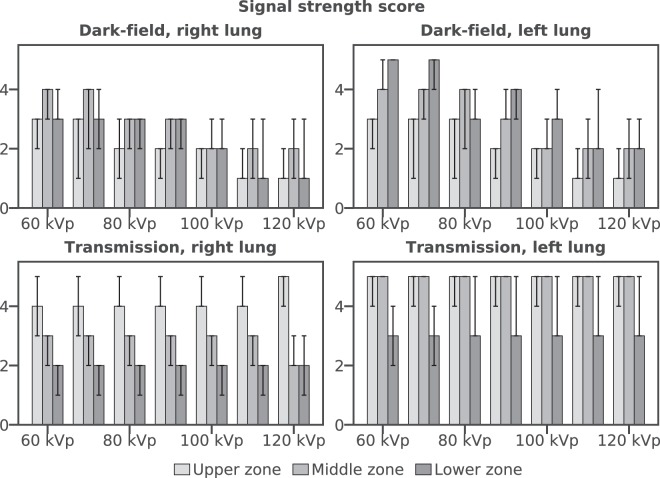
Signal strength score (median and range) of dark-field (top row) and transmission (bottom row) images for the examined tube voltages. Signal strength score in dark-field images increases from the upper left towards the lower left lung, as the projected thickness of the lung increases. In the right lung, this effect is counteracted by an infiltrate in the lower right lung. In every examined zone, signal strength score decreases for increasing tube voltages. In transmission, signal strength score decreases from the upper lungs to the lower lungs. In every examined zone, the score remains nearly constant for all tube voltages.

Signal strength score for dark-field images was higher at lower tube voltages for each zone of the lung. Highest signal strength scores were achieved in the lower zone of the left lung. In the left lung, signal strength decreased from the lower towards the middle zone, and then decreased further towards the upper zone. This again corresponds to the radiographic findings of better ventilation of the left lung, and thus the highest number of ventilated alveoli in the lower zone of the left lung. In the right lung, the highest signal strength score was found for the middle zone, while equal values were achieved in the upper and lower zone. The reduced score in the right-lung lower zone (compared to the left-lung lower zone) corresponds with the infiltrate in this region. Grading results for dark-field and transmission are compiled visually in Fig. 3, as well as in Supplementary Table S1.

**Figure 4:**
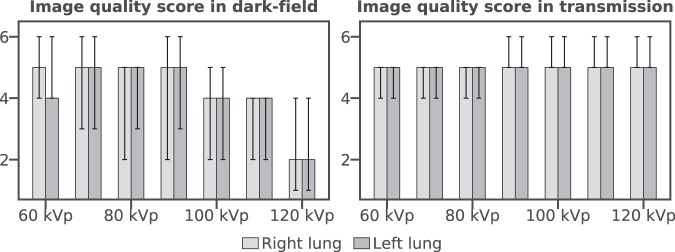
Image quality score (median and range) of dark-field (right) and transmission (left) images for the examined tube voltages. Image quality score decreases for higher tube voltages in dark-field images. In transmission images, the score remains constant.

For transmission radiographs, median of image quality score over both lungs and all readers was 5 at each tube voltage [on an ordinal integer scale ranging from 1 to 6 (=best), cf. Methods], with a trend towards higher values at higher voltages. For dark-field images, a median of 5 was achieved for 60 to 90 kVp, apart from the left lung at 60 kVp with a median of 4. At higher voltages, image quality scores decreased, down to a median score of 2 at 120 kVp (see Fig. 4 and Supplementary Table S2).

High correlation coefficients *r* between the “grouped” and “individual” reading sessions were achieved (Table 1). Here, *r* was greater than 0.8 (representing almost perfect agreement) for each reader regarding dark-field images (apart from reader 1 for image quality) and greater than 0.9 for each reader regarding transmission radiographs (apart from reader 3 for image quality). Some coefficients (denoted N/A) could not be calculated due to zero variance in one reader’s score of transmission image quality.

**Table 1:** Inter-reader agreement (both reading sessions) and agreement for each reader between “grouped” and “individual” reading sessions. Agreement is given in Spearman’s rho. Values of 0.81–1.0 are considered as almost perfect agreement, 0.61–0.80 as substantial agreement, and 0.41–0.60 as moderate agreement. N/A denotes undefined values for Spearman’s rho due to zero variance of one reader’s transmission image quality score.

	Signal strength		Image quality	
	Dark-field	Transmission	Dark-field	Transmission
**Inter-reader agreement (combined from both reading sessions)**				
Readers 1 and 2	0.755	0.623	0.498	N/A
Readers 1 and 3	0.754	0.674	0.310	0.861
Readers 2 and 3	0.658	0.918	0.677	N/A
**Agreement between “grouped” and “individual” reading sessions**				
Reader 1	0.814	0.999	0.473	1.000
Reader 2	0.952	0.980	0.882	N/A
Reader 3	0.945	1.000	0.823	0.730

### Quantitative evaluation of dark-field contrast

**Figure 5:**
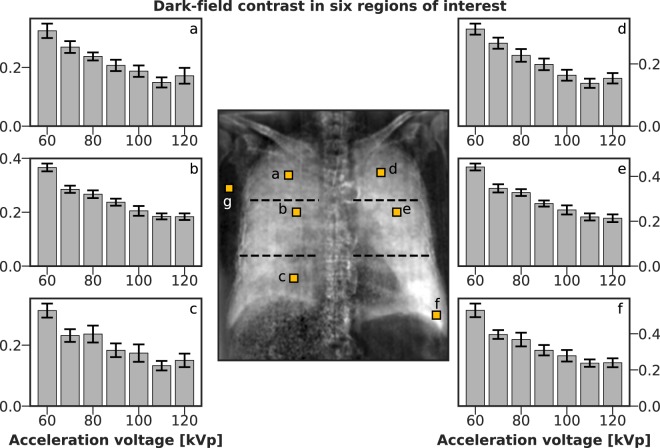
Dark-field contrast as a function of tube voltage in six ROIs (**a**–**f**), with respect to an ROI in the vicinity of the lung (**g**). Each ROI is a 30 × 30 pixel square, placed at highlighted locations in the central overview image. Graphs correspond to ROIs with the same letter. ROIs were placed such that ribs were avoided. Contrast decreases towards higher tube voltages in all lung zones. The horizontal dashed lines in the overview image approximate the extent of the six lung zones assumed in the reader study.

For a region of interest (ROI) in each of the six lung zones, dark-field contrast was calculated with respect to an ROI in the area surrounding the lung (see Fig. 5). Contrast decreases towards higher acceleration voltages for all lung zones, except for a slight deviation from the trend at 120 kVp. This dependence on tube voltage is in agreement with the trend of signal strength score seen in Fig. 3. It is clearly visible for all tube voltages that the highest dark-field contrast is generated in the lower zone of the left lung.

## Discussion

Quantitative evaluation of lung dark-field contrasts showed that the highest contrast was obtained at 60 kVp for all ROIs. Inconsistencies between contrast changes in different ROIs (e.g. contrast increase from 110 to 120 kVp in ROIs a and d, or the discontinuities visible in ROI c) may be partly due to an observed slight expansion of the lung between measurements, despite maintaining a constant ventilation pressure of 25 mbar. In particular, ROI c is located in the area of the infiltrate, which may have been affected by the ventilation of the lung. Duration of the total measurement procedure was approx. 40 min. As lung ventilation may vary over time and between different lung regions, it may account for such local variations. The findings of the quantitative evaluation coincide however with reader study scores for dark-field signal strength and image quality, which are highest at 60 to 70 kVp.

Clinical findings of the body showed that infiltrates were present in the right lung, particularly in the lower zone. This is concordant with subjective and objective evaluation, which identified a dark-field signal decrease in the lower zone of the right lung, compared to the lower zone of the left lung. In the left lung, which shows no infiltrates, a signal strength decrease from the lower towards the upper zone was found. This finding reflects the fact that the amount of alveolar parenchyma (i.e., the number of air-tissue interfaces) traversed by the X-ray beam increases towards the base of the healthy lung. Subjective signal strength of conventional images also reflects the clinical finding of weaker signal (more opacification) in the right lower lung.

The range of X-ray spectra yielding both acceptable conventional radiographs and good dark-field images (with current technology) is narrow: Low-energy X-ray spectra yield low detector dose at acceptable patient dose levels, whereas high-energy X-ray spectra carry little visibility and achieve only a weak dark-field signal from the lung. The lowest examined tube voltage was 60 kVp because the source’s maximum tube current, and hence, maximum achievable entrance surface dose (ESD) rapidly drops for lower voltages. Disregarding technical limitations, the increase of attenuation at lower X-ray energies will also strongly diminish dose efficiency. However, dark-field thorax imaging slightly below 60 kVp may be practical with an X-ray source achieving sufficiently high ESD values at such low voltages. The source’s maximum acceleration voltage (125 kVp) determined the range’s upper limit of 120 kVp. We consider further increases of acceleration voltage unnecessary, as we expect the trend of decreasing signal strength and image quality in the dark-field images to continue.

The constraints for determining optimal tube voltage are not uniquely defined. The most obvious approach would be to apply equal estimates for effective dose (ED) at every tube voltage setting. For radiography, ED can be estimated from dose-area product (DAP). However, the conversion factor between the two is dependent on tube voltage, and conversion factors for thorax radiography are, to our knowledge, not tabulated for most X-ray energies used here. It may be beneficial for future studies to estimate these factors from Monte-Carlo simulations on anthropomorphic phantoms. In this work, estimated ESD values increase with tube voltage (cf. Table 2), but increasing attenuation implies that a greater fraction of this dose is deposited in the body at lower X-ray energies.

**Table 2:** Image acquisition parameters. At every tube voltage one image was acquired. All seven scans were acquired in a time span of 40 min. During each scan, every part of the field of view received 25 × -ray pulses of 20 each. Visibility is given as mean ± standard deviation across the field of view. ESD: entrance surface dose measured at source distance of 152 cm (16 cm above patient table).

Tube voltage [kV]	60	70	80	90	100	110	120
Visibility [%]	36 ± 4	29 ± 3	22 ± 3	19 ± 2	18 ± 2	18 ± 2	18 ± 2
Tube current [mA]	600	360	450	400	360	327	300
ESD [mGy]	0.9	0.8	1.3	1.5	1.5	2.0	2.2

With the exception of signal bias for very low detector doses, mean dark-field signal is dose-independent (whereas noise levels are not). Therefore, we expect that a repetition of the presented measurements with constant effective dose would yield comparable results: the influence of dark-field noise levels was largely eliminated due to the application of the low-pass filter (cf. Methods).

Grating-based X-ray dark-field imaging always yields a simultaneously acquired, perfectly registered conventional X-ray image. It stands to reason that conventional and dark-field X-ray image data should be combined for diagnosis, e.g. for the correlation of dark-field image findings with anatomical structures. Ideally, attenuation data from dark-field radiography could even render a separate conventional radiographic exam unnecessary. However, the determined optimal tube voltage settings for dark-field imaging of the thorax are significantly lower than those typically used for radiography of the lung. Although signal strength and image quality scores of the acquired conventional radiographs show no clear dependence on tube voltage, they are at odds with multiple studies and long-term clinical experience, which place optimum tube voltage for imaging of the lung around 120 to 130 kVp^21,22^. One reason for this discrepancy may be the added filtration due to the three gold gratings, as well as the suppression of detected Compton-scattered radiation due to the gratings and the narrow collimation of the X-ray field.

Taking for granted the current state of technology, there are multiple approaches to combine dark-field and conventional radiography. For example, dark-field radiography might be performed at suitably low X-ray energies, and a separate conventional radiographic exam is performed, while the transmission image from the dark-field setup is used only for correlating dark-field imaging findings with anatomical structures.

Alternatively, a conventional radiograph may not indicated for the examined disease pattern (e.g. early-stage emphysema or fibrosis). Finally, dark-field imaging could be performed at a higher tube voltage, at the expense of dark-field image quality, but then also delivers a diagnostic-quality thorax radiograph. Among other factors, an appropriate decision will depend on: the added diagnostic value of clinical thorax dark-field imaging for various structural lung pathologies (at both low and high X-ray energies), whether or not the increase in patient dose is justified by these improvements, whether a conventional radiographic exam is indicated for the given disease pattern, and whether a low-energy conventional radiograph from a dark-field setup may even be sufficient to replace a standard radiographic exam.

One limitation of the study is that only a single dead body was examined. Although lung dark-field signal strength is probably lower for a dead body than that for a living person, and is expected to depend on subject age and health, these differences are likely dominated by variations in the *amount* of scattering material, not variations in the *energy-dependence* of dark-field signal between subjects’ lungs. Such variations may be present, but are likely to affect the energy-dependence of −ln(DF) much less than its sample-independent proportionality with the inverse square of photon energy. On the other hand, variations in the amount of scattering material will alter dark-field signal levels, but not their trend as a function of X-ray energy. Finally, as dose efficiency at low energies probably decreases for overweight patients, a possible dependence of optimal tube voltage on patient weight is yet to be determined. The examined body was of normal weight (BMI = 24.2). In the current study, only the lung was examined as the thorax is of main interest for dark-field imaging currently. However, in future studies the optimum tube voltage should be examined for other regions or applications if dark-field imaging is also used there.

Signal level and image quality scores showed greater variation for dark-field images than for transmission images. Although readers were experienced in dark-field imaging and received training before the beginning of the study, they can not be as experienced as in transmission radiography as this is done in the daily practice and thus, inter-reader agreement was lower for dark-field imaging compared to transmission radiographs. Nonetheless, high values for inter-reader agreement as well as for intra-reader agreement between the two reading sessions were shown even for dark-field images. Hereby, a number of three readers seems appropriate due to the high correlation values. Thus, apparently dark-field images are rated reliably, regardless of whether images were presented all at once or individually.

Finally, the optimum tube voltage for dark-field is highly dependent on setup-specific parameters, such as grating materials and periods, as well as inter-grating distances. The presented setup uses an unconventional asymmetric geometry (cf. Methods), which provides a more achromatic visibility spectrum than a Talbot-Lau interferometer constructed with similar gratings. Trends for image quality and signal strength may therefore vary for such setups. Performance increases for X-ray dark-field imaging at high X-ray energies might be achieved by increasing setup sensitivity, e.g. by reduction of grating periods or increase of interferometer length. Reduction of grating periods while maintaining constant attenuation capabilities is synonymous to an increase of aspect ratios, which is hampered by major manufacturing challenges. Increasing interferometer length is limited due to the need to fit into clinical examination rooms, the increased demands on mechanical stability, and a requirement for detectors with very large fields of view.

In conclusion, for dark-field radiography of the human thorax at the used setup, both quantitative and subjective grading of signal strength, as well as subjective grading of image quality point to an ideal tube voltage of 60 to 70 kVp. Although some variation in the observed trend may occur due to a different behaviour of dark-field signal in living subjects, as well as differences in setup design, we believe deviations from our results to be limited. Considering the achieved high transmission image quality scores at all tube voltages, the use of transmission images generated from a low-energy (60 to 70 kVp) dark-field setup for clinical imaging should be considered.

## Methods

### Imaging setup

A three-grating fringe-scanning arrangement was used for the acquisition of dark-field radiographs. A schematic of the setup can be found in Supplementary Fig. S4. Three absorption gratings with gold heights ranging from 150 to 250 were arranged in a highly asymmetric configuration [grating periods: *p*(*G*_0_) = 68.72 μm, *p*(*G*_1_) = 8.73 μm, *p*(*G*_2_) = 10 μm, inter-grating distances: *d*(*G*_0_ − *G*_1_) = 1.60 m, *d*(*G*_1_ − *G*_2_) = 0.23 m]. The analyzer grating is therefore much closer to the modulation grating than the Talbot distance for the used X-ray energies, meaning that the Talbot self-imaging effect is not exploited. Propagation effects are sufficiently weak that the “direct” shadow of the modulation grating is analyzed, yielding high visibility for a greater than usual range of X-ray energies^28^.

While a single tile (5 × 5^2^ cm^2^) was used as a source grating, both modulation and analyzer gratings were each assembled from eight individual tiles of 2.5 × 5^2^ cm^2^, yielding a total area of 40 × 2.5^2^ cm^2^ (ref.^29^). The table plane was 5.6 cm above the modulation grating. Setup parameters are also documented in previous publications^16,17^. All gratings were mounted on a common frame to be able to perform fringe-scanning acquisition^30,31^: During acquisition, the frame is rotated on an axis through the source’s focal spot, while the remaining components remain stationary. Thus, a total effective field of view of 32 × 35 cm^2^ is sampled in a scan time of 40s. A high-flux rotating-anode X-ray tube (MRC 0310 ROT GS, Philips Medical Systems, Hamburg, Germany) and a flat-panel radiography detector (Pixium RF 4343, Trixell, Moirans, France) were used. Image acquisition parameters are given in Table 2. Entrance surface dose (ESD, excluding patient backscatter) was measured ahead of time for all acceleration voltages using the “NOMEX Multimeter” (PTW-Freiburg, Freiburg, Germany) and calculated assuming proportionality of ESD with tube current.

### Data acquisition and processing

For scanning the whole field of view, a Moiré fringe-scanning method was used: By induction of a slight mismatch of grating periods to inter-grating distances, local variations in the relative shift of the gratings are introduced, which becomes apparent as spatial variations of intensity (Moiré fringes). Moving a sample through a field of view with such fringes allows sampling the same feature at multiple relative grating shifts, which would more commonly be induced by phase-stepping, i.e. motorized movement of one grating between acquisitions. Data acquired in this manner can therefore be interpreted and processed in a way comparable to phase-stepping, allowing the retrieval of dark-field, transmission and differential phase values of the sample^30,31^. In the present setup, the interferometer is moved on a circular arc around the focal spot during acquisition, while the X-ray source, sample, and detector remain stationary (cf. Supplementary Fig. S4).

Since the visibility is energy-dependent and a polychromatic source is used, visibility reduction due to beam-hardening occurs in the sample. A method for correcting this effect, similar to the one presented by Pelzer *et al*.^32^, was applied. Lastly, dark-field radiographs were low-pass filtered by convolution with a 2D Gaussian kernel (*σ* = 3.2 px). This leads to a reduction of standard deviation by a factor of ≈12 (for the simplified assumption that input data consists of normally-distributed pixel values with constant mean and variance). This is intended to allow a clearer presentation of relevant image features: The high noise levels entirely obscure small features in the dark-field images, while also creating a visual impression unsuitable for radiographic diagnosis. Filtering reduces noise levels to approximately those of ordinary radiography, while no (previously discernable) image features are lost. Among a series of kernel sizes, the presented one was previously found to strike an acceptable balance between visual impression and spatial resolution. No filtering was applied to the conventional radiographs.

### Human body

The experiment was approved by the institutional review board (Ethikkommission der Ludwig-Maximilians-Universität München, Pettenkoferstr. 8a, 80336 München, project number 14–13) and was conducted according to the Declaration of Helsinki. The body of a 64-year-old male was 169 cm in height and weighed 69 kg. Measurements were performed 48 h post mortem, in supine anteroposterior position. During the measurements, the lung was inflated with 25 mbar. In an examination by the coroner, a possible infiltrate in the lung was identified.

### Reader Study

Subjective image analysis was performed independently by three blinded radiologists with 3, 10 and 10 years experience (F.M., D.P., A.A.F.). All readers were familiar with dark-field images as they participated in previous studies and are involved in the scientific field of dark-field imaging. However, they received training before the start of the study as dark-field imaging is not yet established in clinical routine. In this reading session, images with low, moderate and high signal strength were presented as a reference. Additionally, images of previous animal studies were demonstrated to illustrate the influence of pathologies such as fibrosis or pneumothorax. In a first reading session, each image was rated individually and in a second session, all images of one type (dark-field or transmission) were shown grouped for a direct comparison of the different tube voltages. In each reading session, signal strength and image quality were evaluated. Signal strength for dark-field images and opacification for transmission images were rated on a six-point scale, respectively. Window settings were fixed for an ideal comparison without influence of individual windowing. For dark-field images, levels were: 0 - no signal; 1 - low signal; 2 - low-moderate signal; 3 - moderate signal; 4 - moderate-high signal; 5 - high signal. For transmission images, levels were: 0 - complete consolidation with no lung visible (e.g. effusion); 1 - consolidation (e.g. lobar pneumonia); 2 - between 1 and 3; 3 - ground glass; 4 - between 3 and 5; 5 - normal lung. Images were divided into six zones: right/left lung, upper/middle/lower zone (see also Fig. 5). Each zone was evaluated individually regarding signal strength/opacification. Image quality was also rated on a six-point scale: 1 - not diagnostic; 2 - sufficient; 3 - satisfactory; 4 - good; 5 - very good; 6 - excellent. The same scale was used for dark-field and transmission images. Image quality was evaluated separately for the left and the right lung. Here, window levels were not fixed and could be changed by the readers for obtaining an individually optimal image impression.

### Statistical analysis

Statistical analysis was performed by dedicated software packages (SPSS, IBM, USA; Excel 2016, Microsoft, USA; Prism 7, Version 7.0c, USA). Continuous data are expressed as arithmetic mean ± standard deviation. Statistical evaluation of subjective image criteria was performed using Wilcoxon signed-rank test. A p-value ≤ 0.05 was considered to indicate statistical significance. Inter-reader agreement and agreement between the two reading sessions were evaluated by using Spearman’s rho, as data did not show Gaussian distribution. Values <0 were regarded as indicating no agreement and 0–0.20 as slight, 0.21–0.40 as fair, 0.41–0.60 as moderate, 0.61–0.80 as substantial, and 0.81–1 as almost perfect agreement^33^.

### Quantitative evaluation of dark-field signal

Dark-field signal DF is calculated from visibility reduction *ν* = *V*_s_/*V*_r_ (where *V*_s_ and *V*_r_ are sample and reference visibilities) and visibility reduction *ν*_BH_ due to beam-hardening via: DF = −ln(*ν*/*ν*_BH_). An estimate for *ν*_BH_ was calculated from X-ray transmittance with an algorithm similar to the one presented by Pelzer *et al*.^32^.

For quantitative evaluation, six ROIs (a–f) were selected in the lung, one per lung zone. A seventh ROI (g) was defined in the vicinity of the lung to determine background signal levels. Each ROI is a 30 × 30 pixel square, corresponding to 11 × 11 mm^2^ in the plane of the sample table. The size was chosen to allow placement of ROIs between ribs, and thereby exclude them from the analysis. Thorax movement between image acquisitions due to ventilation (e.g. continued expansion of the thorax) was minimal, so that every ROI coincided with the same anatomical region for each tube voltage. Mean values *μ*_*r*,*U*_ and standard deviations *σ*_*r*,*U*_ were calculated over each ROI *r* at every tube voltage *U* = 60, 70, …, 120 kVp:

$${\mu }_{r,U}={\langle {\rm{DF}}(U)\rangle }_{r},\,{\sigma }_{r,U}=\sqrt{{{\rm{Var}}}_{r}[{\rm{DF}}(U)]}\,(r=a,\ldots ,g\mathrm{).}$$

Dark-field contrasts *C* as presented in Fig. 5 were defined as the difference of the means over lung and background ROI. Their standard deviations *σ*^(*C*)^ (error bars in Fig. 5) were calculated accordingly:

$${C}_{r,U}={\mu }_{r,U}-{\mu }_{g,U},\,{\sigma }_{r,U}^{(C)}=\sqrt{{\sigma }_{r,U}^{2}+{\sigma }_{g,U}^{2}};\,(r=a,\ldots ,f\mathrm{).}$$

## Supplementary information

Supplementary Tables and Figures
