# Supplementary material for: Optimization of tube voltage in X-ray dark-field chest radiography
Source: Sci Rep. 2019 Jun 18;9:8699. doi: 10.1038/s41598-019-45256-2 (PMC6582156; doi:10.1038/s41598-019-45256-2)
Supplement: Supplementary file 1 — Supplementary Tables and Figures [file 41598_2019_45256_MOESM1_ESM.pdf]

Optimization of tube voltage in X-ray dark-field chest radiography – Supplementary Information

Andreas P. Sauter[1,+,*], Jana Andrejewski[2,+], Fabio De Marco[2], Konstantin Willer[2], Lukas B. Gromann[2], Wolfgang Noichl[2], Fabian Kriner[3], Florian Fischer[3], Christian Braun[3], Thomas Koehler[4], Felix Meurer[1], Alexander A. Fingerle[1], Daniela Pfeiffer[1], Ernst Rummeny[1], Julia Herzen[2], and Franz Pfeiffer[1,2]

[1]Department of Diagnostic and Interventional Radiology, Technical University of Munich, 81675 Munich, Germany.
[2]Chair of Biomedical Physics, Department of Physics and Munich School of BioEngineering, Technical University of Munich, 85748 Garching, Germany.
[3]Institut für Rechtsmedizin, Ludwig-Maximilians-Universität München, 80336 Munich, Germany.
[4]Philips GmbH Innovative Technologies, Research Laboratories, 22335 Hamburg, Germany.
*andreas.sauter@tum.de
+these authors contributed equally to this work

Signal strength score

	Dark-field, individual readings						Transmission, individual readings					
kVp	R-UZ	R-MZ	R-LZ	L-UZ	L-MZ	L-LZ	R-UZ	R-MZ	R-LZ	L-UZ	L-MZ	L-LZ
60	2, **3**, 3	3, **4**, 4	3, **3**, 4	2, **3**, 3	4, **4**, 5	5, **5**, 5	3, **4**, 5	2, **3**, 3	1, **2**, 2	4, **5**, 5	5, **5**, 5	2, **3**, 4
70	1, **3**, 3	2, **4**, 4	2, **3**, 4	1, **3**, 3	3, **4**, 4	4, **5**, 5	3, **4**, 5	2, **3**, 3	1, **2**, 2	4, **5**, 5	5, **5**, 5	2, **3**, 4
80	1, **2**, 3	2, **3**, 3	2, **3**, 3	1, **3**, 3	2, **4**, 4	3, **3**, 4	4, **4**, 5	2, **3**, 3	1, **2**, 2	4, **5**, 5	5, **5**, 5	3, **3**, 5
90	1, **2**, 2	2, **3**, 3	2, **3**, 3	1, **2**, 2	3, **3**, 4	3, **4**, 4	4, **4**, 5	2, **3**, 3	1, **2**, 2	4, **5**, 5	5, **5**, 5	3, **3**, 5
100	1, **2**, 2	2, **2**, 3	2, **2**, 3	1, **2**, 2	2, **2**, 3	3, **3**, 4	4, **4**, 5	2, **3**, 3	1, **2**, 2	4, **5**, 5	5, **5**, 5	3, **3**, 5
110	1, **1**, 2	1, **2**, 3	1, **1**, 3	1, **1**, 2	1, **2**, 3	2, **2**, 4	4, **4**, 5	2, **3**, 3	1, **2**, 2	4, **5**, 5	5, **5**, 5	3, **3**, 5
120	1, **1**, 2	1, **2**, 3	1, **1**, 3	1, **1**, 2	1, **2**, 3	2, **2**, 3	4, **5**, 5	2, **2**, 3	1, **2**, 3	4, **5**, 5	5, **5**, 5	3, **3**, 5
	Dark-field, grouped readings						Transmission, grouped readings					
kVp	R-UZ	R-MZ	R-LZ	L-UZ	L-MZ	L-LZ	R-UZ	R-MZ	R-LZ	L-UZ	L-MZ	L-LZ
60	2, **2**, 2	3, **3**, 4	2, **3**, 3	2, **3**, 3	3, **4**, 4	5, **5**, 5	3, **4**, 5	2, **3**, 3	1, **2**, 2	4, **5**, 5	5, **5**, 5	2, **3**, 4
70	1, **2**, 3	2, **3**, 4	2, **3**, 3	1, **3**, 3	3, **4**, 4	4, **5**, 5	3, **4**, 5	2, **3**, 3	1, **1**, 2	4, **5**, 5	5, **5**, 5	2, **3**, 4
80	1, **2**, 2	2, **3**, 3	2, **3**, 3	1, **2**, 2	2, **3**, 3	3, **4**, 4	4, **4**, 5	2, **3**, 3	1, **2**, 2	4, **5**, 5	5, **5**, 5	3, **3**, 5
90	1, **2**, 2	2, **3**, 3	2, **3**, 3	1, **2**, 2	2, **3**, 3	3, **4**, 4	4, **4**, 5	2, **3**, 3	1, **2**, 2	4, **5**, 5	5, **5**, 5	3, **3**, 5
100	1, **1**, 2	2, **2**, 2	2, **2**, 2	1, **1**, 2	2, **2**, 2	3, **3**, 3	4, **4**, 5	2, **3**, 3	1, **2**, 2	4, **5**, 5	5, **5**, 5	3, **3**, 5
110	1, **1**, 2	1, **2**, 2	1, **2**, 2	1, **1**, 2	1, **2**, 3	2, **2**, 3	4, **4**, 5	2, **3**, 3	1, **2**, 2	4, **5**, 5	5, **5**, 5	3, **3**, 5
120	1, **1**, 1	1, **2**, 2	1, **1**, 1	1, **1**, 1	1, **2**, 2	2, **2**, 2	4, **5**, 5	2, **3**, 3	1, **2**, 2	4, **5**, 5	5, **5**, 5	3, **3**, 5

Supplementary Table S1. Signal strength score of dark-field and transmission images (as derived from the reader study) at all examined tube voltages. Values for the individual readings as well as for the grouped readings are shown. Readings were performed for three zones of every lung, resulting in a total of six zones for every image [right/left lung: R/L, upper/middle/lower zone: UZ/MZ/LZ]. Value triplets (one per reader) are given as: minimum, median (bold), maximum.

Image quality score

	Individual readings				Grouped readings			
	Dark-field		Transmission		Dark-field		Transmission	
kVp	R	L	R	L	R	L	R	L
60	4, **5**, 6	4, **4**, 6	4, **5**, 5	4, **5**, 5	4, **5**, 5	4, **4**, 5	4, **5**, 5	4, **5**, 5
70	3, **5**, 6	3, **5**, 6	4, **5**, 5	4, **5**, 5	3, **5**, 5	3, **5**, 5	4, **5**, 5	4, **5**, 5
80	2, **5**, 5	3, **5**, 5	4, **5**, 5	4, **5**, 5	2, **5**, 6	3, **5**, 6	4, **5**, 6	4, **5**, 6
90	2, **5**, 6	3, **5**, 6	5, **5**, 6	5, **5**, 6	2, **5**, 5	2, **5**, 5	5, **5**, 6	5, **5**, 6
100	2, **4**, 5	2, **4**, 5	5, **5**, 6	5, **5**, 6	2, **3**, 5	3, **3**, 5	5, **5**, 6	5, **5**, 6
110	2, **4**, 4	2, **4**, 4	5, **5**, 6	5, **5**, 6	2, **2**, 5	2, **3**, 5	5, **5**, 6	5, **5**, 6
120	1, **2**, 4	1, **2**, 4	5, **5**, 6	5, **5**, 6	1, **2**, 4	1, **2**, 4	5, **5**, 6	5, **5**, 6

Supplementary Table S2. Subjective image quality score (as derived from the reader study) of dark-field and transmission images for the right (R) and the left (L) lung at the different tube voltages. Values for the individual readings, as well as for the grouped readings are shown. Value triplets (one per reader) are given as: minimum, median (bold), maximum.

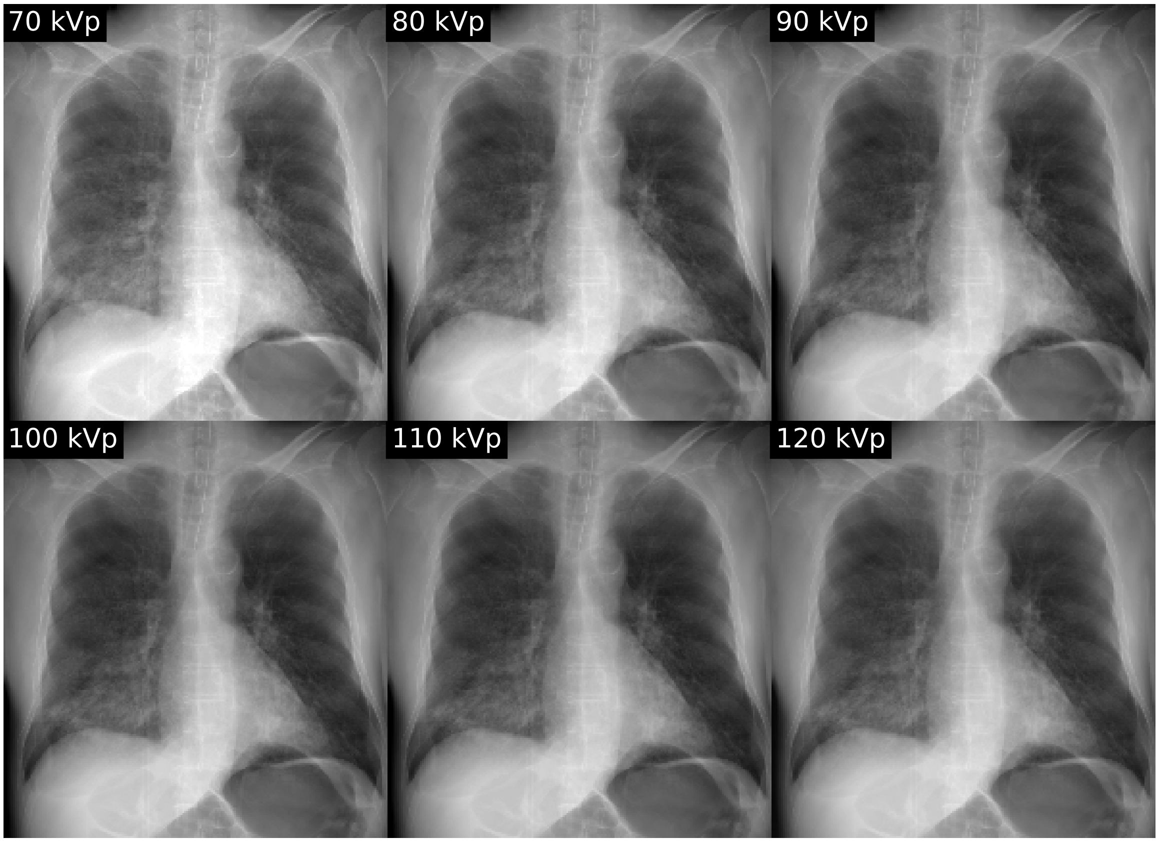

Supplementary Figure S3. Transmission radiographs of a human thorax at 70 to 120 kVp. These images and the one taken with 60 kVp (main article, Fig. 1) are shown with the same windowing. Signal strength in the lung barely changes for different tube voltages.

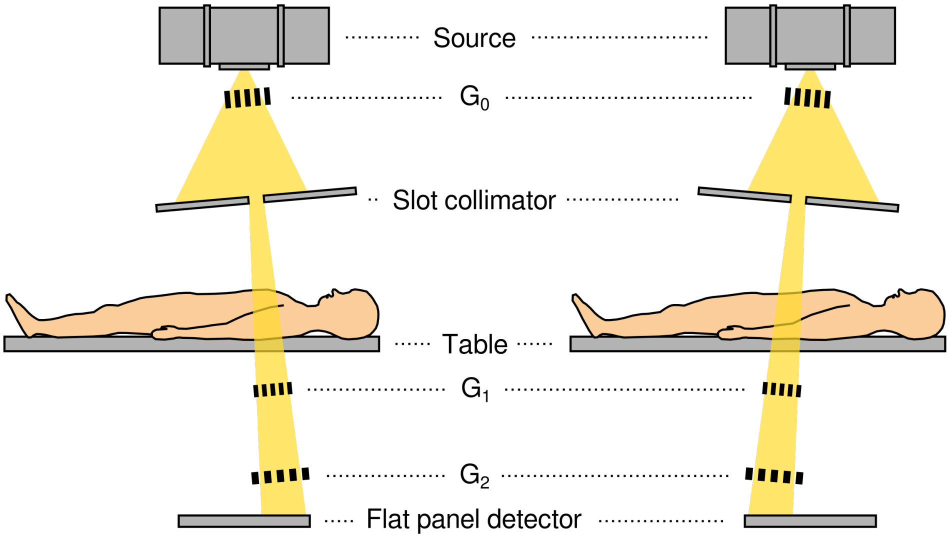

Supplementary Figure S4. Schematic of the large-field-of-view X-ray dark-field chest imaging system. All three gratings and a slot collimator between G_0 and the sample table are fixed on a common frame, which is rotated around an axis through the focal spot during fringe-scanning acquisition. Imaged subject, source, and detector remain stationary during the measurement. Figure adapted from Gromann et al. (cf. Ref 16) according to 'CC BY 4.0' (https://creativecommons.org/licenses/by/4.0/).
